# A Rare Granular Cell Tumor with a Center Ulcer of the Rectum

**DOI:** 10.1155/2020/8563718

**Published:** 2020-01-13

**Authors:** Jeonghun Lee, Younghye Kim

**Affiliations:** ^1^Department of Internal Medicine, Ye Hospital, Anyang, Republic of Korea; ^2^Green Cross Reference Laboratory, Yongin, Republic of Korea

## Abstract

We report a very rare case of granular cell tumor with a center ulcer of the rectum, which was detected in a 47-year-old man during screening colonoscopy. It is difficult to distinguish granular cell tumor from other subepithelial tumors. However, our case had a center ulcer unlike previous cases. We confirmed the diagnosis using histological and immunohistochemical examinations. In addition to our case report, we discuss the morphology, size, layer of invasion, and treatment of rectal granular cell tumors based on a literature review.

## 1. Introduction

Granular cell tumor (GCT) is a soft tissue neoplasm that arises from Schwann cells. It is usually benign and most commonly found in the skin, subcutaneous tissue, oral cavity, and gastrointestinal tract [1]. About less than 11% of GCTs are incidentally found in the gastrointestinal tract, where they are most commonly found in the esophagus, followed by the duodenum and stomach [2]. Rectal involvement is extremely rare. There are very few reported cases of GCT of the rectum, which usually present a sessile polypoid lesion which is smooth and covered with normal mucosa. We report a very rare case of rectal GCT with a center ulcer in a 47-year-old man and compare our findings with those of previous reports.

## 2. Case Report

**Figure 1:**
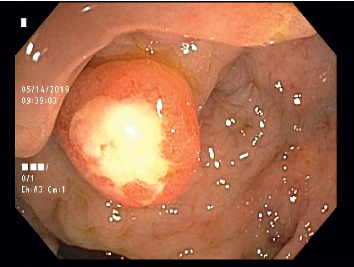
A 10 mm pedunculated polyp with a central ulceration was found in the rectum.

**Figure 2:**
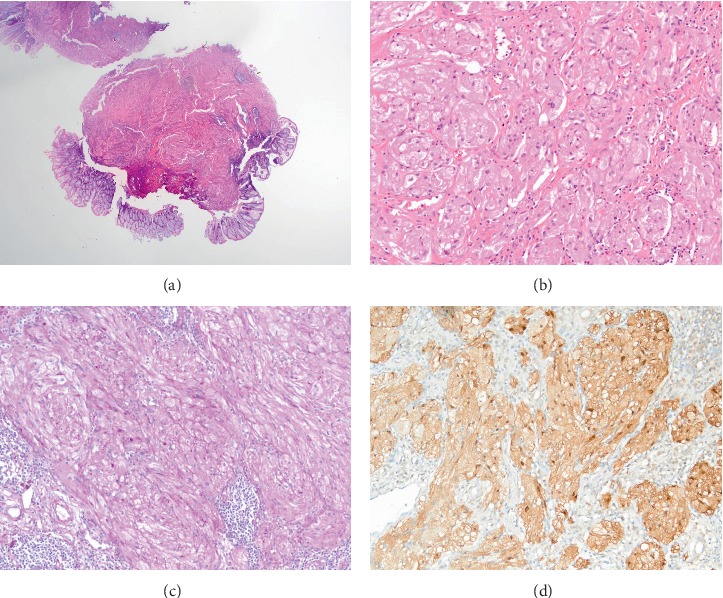
Histopathologic features of a rectal granular cell tumor. (a) The tumor was seen in the submucosa (hematoxylin and eosin staining, ×12.5). (b) The tumor cells showed a polygonal or spindle-shaped cytoplasm with mild-to-moderate nuclear atypia and abundant eosinophilic granules (hematoxylin and eosin staining, ×200). (c) Immunohistopathology staining with periodic acid-Schiff was positive (original magnification, ×200). (d) Immunohistopathology staining for S-100 protein was positive (original magnification, ×200).

A 47-year-old man presented to our hospital for screening colonoscopy. He had no remarkable medical history or gastrointestinal symptom. A 10 mm reddish pedunculated polypoid lesion with a center ulcer was found in the rectum, 5 cm from the anus (Figure 1). Based on the macroscopic appearance, a tubular adenoma and a carcinoid tumor were included in the differential diagnosis. Although not examined by endoscopic ultrasonography (EUS), the lesion was removed via an endoscopic procedure. Pathologic results showed that the tumor invasion was restricted to the submucosa layer (Figure 2(a)). Tumor cells showed a polygonal or slightly spindle-shaped cytoplasm with mild-to-moderate nuclear atypia and eosinophilic and fine cytoplasmic granules (Figure 2(b)). The cytoplasmic granules had a positive reaction to periodic acid-Schiff staining (Figure 2(c)). Immunohistochemical staining for S-100 protein was also positive (Figure 2(d)). Based on these results, GCT of the rectum was diagnosed. Colonoscopy was performed 3 months later, and no recurrence was observed.

## 3. Discussion

GCT, also called Abrikossoff tumor or granular cell myoblastoma, was first described by Abrikossoff in 1926 [3]. Colonic involvement is rare, and rectal involvement is extremely rare.

Colorectal GCT occurs predominantly between the ages of 30 and 50 years [4]. It is found incidentally during screening colonoscopy and tends to be asymptomatic. On white-light colonoscopy, it usually presents as a solitary sessile polypoid lesion, measuring less than 2 cm in diameter, which is smooth and covered with normal mucosa. Because finding a “molar-shaped” tumor with a central depression, as seen in esophageal GCT, is rare and the endoscopic ultrasonographic findings of GCT are similar to those of other subepithelial tumors (SETs), it is difficult to distinguish GCTs from other SETs, especially carcinoid tumor [5]. The definitive diagnosis of GCT is based on histopathologic findings and positive staining for S-100 protein.

There are only a few studies on GCT of the rectum. We searched on PubMed for cases related to rectal GCT, which were published after the year 2000, using the phrase “granular cell tumor and rectum.” We found only six case reports and 13 patients (Table 1) [1, 5–9]. Rectal GCT was more common in men than in women (ratio 12 : 2; including our patient). However, its sex distribution remains unclear because of its rarity [2]. Their average age was 51.7 years. Most affected patients were asymptomatic, and the tumors were identified incidentally. However, in a report from Japan, five of the eight patients had a positive fecal occult blood test. This was probably due to the regular medical check-up system in Japan. Most of the tumors were benign and were removed via endoscopic procedures. Furthermore, most of the rectal GCTs were smooth and covered with normal mucosa, similar to SET. However, our case involved a reddish pedunculated lesion with an ulceration at the top unlike previous cases. This was probably a secondary change due to an inflammatory reaction to the mucosa, and such lesions are reported in various tumors. Therefore, endoscopists should consider that rectal GCTs may not be similar to SETs, especially carcinoid tumor.

Most GCTs are benign and are fortuitously found. However, less than 2% of them can be malignant [1]. If the tumor size is greater than 4 cm and there is ulceration, cellular necrosis, spindling, pleomorphism, increased nuclear-cytoplasmic ratio, large nucleoli, and increased mitotic activity, the potential for malignancy will be high [7, 10]. Of the factors above, tumor size is the most important factor [11]. Malignant GCTs tend to metastasize, recur, and have a poor prognosis. Therefore, biopsy, EUS, and EUS-guided fine needle aspiration may be valuable for diagnosis. Benign colorectal GCTs that are smaller than 2 cm can be treated via endoscopic resection. Follow-up colonoscopy should be considered valuable as a surveillance modality.

The colonoscopic findings are similar to those of the SETs, but can vary. It is important to analyze the size of the tumor and other features before its treatment because of the possibility of malignancy; most rectal GCTs can be cured via endoscopic resection. Endoscopists should identify the features of GCTs and consider follow-up surveillance via colonoscopy.

## Figures and Tables

**Table 1:** Patient clinical characteristics from the literature.

Case (no)	Age, years	Sex	Signs and symptoms	Morphology	Size, mm	Layer of invasion	Treatment
1^1^	61	M	None	Normal mucosa, sessile	4	N/A	Endoscopic resection
2^5^	49.9 (mean)	6 M/2 F	None: 1, positive FOBT: 5, abdominal discomfort: 2	Yellowish-white: 7; white: 1, all normal mucosa, sessile: 5, pedunculated: 3	<10: 7; 15: 1	Submucosa: most	Endoscopic resection: 5; surgery: 3
3^6^	51	M	None	Yellowish, normal mucosa, sessile	20	Subserosa	Surgery
4^7^	59	M	None	Normal mucosa, sessile	8	Submucosa	Endoscopic resection
5^8^	49	M	N/A	N/A	8	N/A	Endoscopic resection
6^9^	45	M	N/A	N/A	N/A	N/A	N/A
Present case	47	M	None	Reddish, ulcerated, pedunculated	10	Submucosa	Endoscopic resection

M, male; F, female; FOBT, fecal occult blood test; N/A, not available.
